# Supplemental Control of Lepidopterous Pests on Bt Transgenic Sweet Corn with Biologically-Based Spray Treatments

**DOI:** 10.1673/031.009.0801

**Published:** 2009-03-19

**Authors:** Robert R. Farrar, B. Merle Shepard, Martin Shapiro, Richard. L Hassell, Mark. L. Schaffer, Chad. M. Smith

**Affiliations:** ^1^ USDA, Agricultural Research Service, Invasive Insect Biocontrol and Behavior Laboratory, Bldg. 011A, BARC-West, Beltsville, MD 20705, USA; ^2^ Clemson University, Coastal Research and Education Center, 2700 Savannah Highway, Charleston, SC 29414, USA

**Keywords:** Fall armyworm, *Spodoptera frugiperda*, corn earworm, *Helicoverpa* zea, Zea *mays*, *Bacillus thuringiensis*, nucleopolyhedrovirus, nematode, *Beauveria*, neem, spinosad

## Abstract

Biologically-based spray treatments, including nucleopolyhedroviruses, neem, and spinosad, were evaluated as supplemental controls for the fall armyworm, *Spodoptera frugiperda* (J. E. Smith), and corn earworm, *Helicoverpa zea* (Boddie) (Lepidoptera: Noctuidae), on transgenic sweet corn, *Zea mays* (L.) (Poales: Poaceae), expressing a Cry1Ab toxin from *Bacillus thuringiensis* Berliner (Bacillales: Bacillaceae) (Bt). Overall, transgenic corn supported lower densities of both pests than did nontransgenic corn. Control of the fall armyworm was improved in both whorl-stage and tassel-stage corn by the use of either a nucleopolyhedrovirus or neem, but the greatest improvement was seen with spinosad. Only spinosad consistently reduced damage to ears, which was caused by both pest species. In general, efficacy of the spray materials did not differ greatly between transgenic and nontransgenic corn.

## Introduction

The fall armyworm, *Spodoptera frugiperda* (J. E. Smith) (Lepidoptera: Noctuidae) , is a common pest of corn, *Zea mays* (L.), and other crops in the warmer parts of the Americas (Sparks 1979). On corn, it attacks all above ground parts of the plant. It overwinters in southern Florida where it infests sweet corn throughout the growing season (Janes 1973; Foster 1989). Further north, where *S. frugiperda* does not survive winters, it occurs later in the growing season (Hoffman et al. 1996). It can infest whorl stage corn as early as April in coastal South Carolina, where the present study was conducted.

The corn earworm, *Helicoverpa zea* (Boddie) (Lepidoptera: Noctuidae), is also a major pest of corn, as well as numerous other crops (Neunzig 1969; Fitt 1989). It may attack corn in the whorl stage, but does most of its damage by direct feeding on ears. This pest attacks silking corn as early as May in coastal South Carolina.

Transgenic crop plants expressing insect-specific toxins derived from *Bacillus thuringiensis* Berliner (Bacillales: Bacillaceae) (Bt) are important management tools for a variety of lepidopterous and coleopterous pests (Jenkins 1999; Khetan 2001; Kumar 2003). Field corn that expresses a Cry1Ab δ-endotoxin is typically highly resistant to the corn earworm, but is less resistant to the fall armyworm (Abel and Pollan 2004; Chilcutt et al. 2006, 2007). Substantial damage by the corn earworm to transgenic field corn can sometimes occur, however (Chilcutt et al. 2006, 2007). Sweet corn has also been transformed with Cry1Ab genes. As with field corn, transgenic sweet corn is less resistant to the fall armyworm than to the corn earworm (Lynch et al. 1999a, b; Farrar et al. 2004). Corn earworm densities are typically reduced on transgenic sweet corn, but they are not eliminated (e.g., Sorensen and Holloway 1999 e.g., Sorensen and Holloway 2001a, b; Edelson and Mackey 2006). Thus, there is the possibility that transgenic sweet corn may need supplemental control of the fall armyworm as well as the corn earworm, particularly under high pest pressure. Biologically-based materials offer an environmentally benign alternative to conventional chemical pesticides for supplemental control.

Nucleopolyhedroviruses (NPVs) are naturally occurring viruses to which many sawflies and caterpillars, including the fall armyworm and corn earworm, are susceptible. NPVs are usually highly host-specific and have not been shown to be harmful to beneficial insects, the environment, or humans (Gröner 1986, 1990). Fall armyworm NPV, the *S. frugiperda* multiply-embedded nucleopolyhedrovirus (SfMNPV), can be an important factor in the population dynamics of the fall armyworm in the field (Fuxa 1982; Fuxa and Geaghan 1983). Some NPVs, including the *H. zea* singly-embedded nucleopolyhedrovirus (HzSNPV), are commercially available for use as insect control agents. SfMNPV, however, has not been commercialized in the United States. In laboratory tests, fall armyworm larvae consumed less foliage of sweet corn expressing a Cry1Ab toxin than they did of nontransgenic sweet corn foliage. When larvae were fed foliage treated with SfMNPV, reduced rates of consumption led to lower rates of virus-induced mortality on transgenic sweet corn relative to those on nontransgenic sweet corn (Farrar et al. 2004, 2005).

Extracts from the neem tree, *Azadirachta indica* A. Juss. (Sapindales: Meliaceae), are active against hundreds of pest species of several insect orders, while having limited effects on nontarget species, including vertebrates and beneficial insects. The primary active component of neem, azadirachtin, acts as an insect growth regulator as well as a feeding deterrent (Isman 1999; Singh and Saxena 1999; Walter 1999). Jacobson et al. (1984) reviewed neem research within the U.S. Department of Agriculture and reported that the amount of azadirachtin in neem formulations ranged from 0.74 to 2.8 µg/10 µl. The 20 species of insect pests that they tested either died directly from feeding on neem-treated diet or were deterred from feeding. Neem-based insecticides are commonly used in parts of Africa and Asia, and are also commercially available in the United States.

Spinosyns are a group of compounds produced in fermentation by the actinomycete *Saccharopolyspora spinosa* Mertz and Yao (Actinomycetales: Pseudonocardiaceae). These compounds are active against a variety of insects and mites, but particularly against Lepidoptera, and have very low mammalian toxicity (Sparks et al. 1999). The extract of fermentation broth containing a mixture of spinosyns is known as spinosad; spinosad-based insecticides are commercially available and are widely used.

The present study was undertaken to evaluate a wide range of biologically-based tactics as supplemental controls for the fall armyworm and the corn earworm on Bt transgenic sweet corn, and to extend previous laboratory work on NPVs (Farrar et al. 2004, 2005) to the field. Therefore, while the primary focus was on NPVs, a number of other, commercially available, materials were also included. In addition to the aforementioned neem and spinosad, entomopathogenic nematodes (*Steinernema* spp.) (Rhabditida: Steinernematidae), an entomopathogenic fungus (*Beauveria bassiana* (Balsamo) Vuillemin) (Hypocreales: Clavicipitaceae), and a sprayable Bt formulation were included in some tests.

## Materials and Methods

### Plant materials

Seeds of two genotypes of sweet corn, one, GSS 0966 ‘Attribute’ (“Bt corn”), that constitutively expresses a Cry1Ab δ-endotoxin of *B. thuringiensis* subsp. *kurstaki*, and the other its nontransgenic near isoline, cv. ‘Prime Plus’ (“parent corn”), were obtained from Syngenta (www.syngenta.com).

### Spray materials

The SfMNPV used in this study was originally isolated from fall armyworm larvae collected from the field in Georgia (USA) by J. J. Hamm (USDA-ARS, Tifton, GA, USA). For the tests reported herein, it was produced in our laboratory in fall armyworm larvae as described by Farrar et al. (2004, 2005) and prepared as an aqueous suspension containing 2 × 10^9^ occlusion bodies/ml. HzSNPV (Gemstar® LC, 2 × 10^9^ occlusion bodies/ml), neem extract (Neemix® 4.5, liquid, 4.5% azadirachtin), sprayable *B. thuringiensis* (CryMax® WDG, 40% Bt solids, spores, and toxins), and the nematode *Steinernema riobrave* (Cabanillas, Poinar and Raulston) (Biovector 355®, 3.83 × 105 infective juveniles/g) were obtained from Certis USA (www.certisusa.com). Spinosad (SpinTor® 2SC, 22.8% spinosad) was obtained from Dow Agrosciences (www.dowagro.com); *B. bassiana* (Botanigard® ES, 2 × 1011 conidia/g), from Bioworks (www.bioworksinc.com); and the nematode *S. feltiae* (Filipjev) (Scanmask®, 2.5 × 107 infective juveniles per package in a sponge), from BioLogic (www.biologicco.com).

### Field plots

Field plots were established each year from 2002 through 2006 at the Coastal Research and Education Center, Clemson University, Charleston, SC, USA. They were planted relatively late in the season (Table 1) to ensure infestations of both fall armyworms and corn earworms. A split plot design was used, with half of each plot planted with Bt corn and half with parent corn. The plot size was 6 m by 4 rows, spaced 1 m apart, with two guard rows of parent corn on either side of each plot and 1.5 m bare ground buffer between plots within rows to protect against spray drift. These plots were replicated four times, except in 2002, when they were replicated six times.

### Applications

Sprays were applied as “whorl stage” and “tassel stage” treatments. Herein, whorl stage refers to the period before most tassels appeared, while tassel stage refers to the period from that point until harvest. All treatments were applied using a CO2-powered backpack sprayer in a spray volume of 400 liters/ha. (Recommended volumes for foliar applications of insecticides for fall armyworm control on sweet corn are 479 to 718 liters/ha.) Whorl stage treatments were applied by spraying directly over the whorl with one nozzle. Tassel stage treatments were applied using a three-nozzle configuration, with one nozzle over the row and two nozzles directed at the silks. The same volume was used for both stages; the sprayer was calibrated to compensate for nozzle differences.

All sprays were applied as aqueous solutions or suspensions. Application rates of each material, dates, and frequencies and numbers of applications are summarized in Table 1. Joint Venture® (Helena Chemical, www.helenachemical.com), a spreader/sticker marketed for use with biological insecticides, was included in all treatments at 0.125% (vol/vol). Controls were sprayed with water and Joint Venture only.

### Sampling

At the end of the whorl stage, destructive samples for fall armyworms were collected. Ten plants were cut from one row of each plant genotype in each plot. The plants were dissected, fall armyworm larvae were counted, and percentage of plants that were damaged was recorded. Plant height was also recorded at this time, except in 2004.

When the corn reached maturity (milk stage, approximately two months after planting), all ears in one row of each plot, which had not been destructively sampled, were harvested. The number of ears was recorded, and the ears were inspected and data were recorded for numbers of corn earworm and fall armyworm larvae, as well as damage to the ears.

### Statistical analyses

All data were subjected to factorial analysis of variance (ANOVA) by the PROC GLM procedure (SAS Institute 2006). Separate analyses were done for each year. For whorl stage data, dependent variables included plant height (except in 2004), percent damaged plants (except for 2002, when percent of plants with live larvae was used instead), and fall armyworm larvae (all sizes) per plant. For harvest data, dependent variables included ears per sample (6 m of row), percent damaged ears, fall armyworm larvae per ear, and corn earworm larvae per ear. Percentages were arcsine transformed before the analysis, but means presented are of back-transformed data. Independent variables included corn genotype and spray treatment. The effect of the interaction of spray treatment by corn genotype was also tested. When the interaction was not significant (*P* > 0.05), means were separated by the least significant difference (LSD) test (PROC GLM procedure; SAS Institute 2006) for both corn genotypes together. When the interaction was significant, separate analyses of spray treatment effects, with separate LSDs, were performed for each corn genotype. (Separate analyses were also performed for ears per sample in 2004, where *P* = 0.0503 for the interaction.) Differences, increases, or decreases reported below refer to comparisons where *P* ≤ 0.05.

## Results

Results from years 2002 through 2006 are summarized in Tables 2 through 6, respectively. Across spray treatments, there were fewer fall armyworms per whorl stage plant of Bt corn than of parent corn in 2003, 2004, and 2006. Relative to nontransgenic corn, Bt corn had fewer fall armyworms per ear in 2005 and 2006 and fewer corn earworms per ear in all years except 2003. Damage to whorl stage plants was reduced on Bt corn in all years in which it was measured, while damage to ears of Bt corn was reduced in all years except 2004.

**Table 1.**
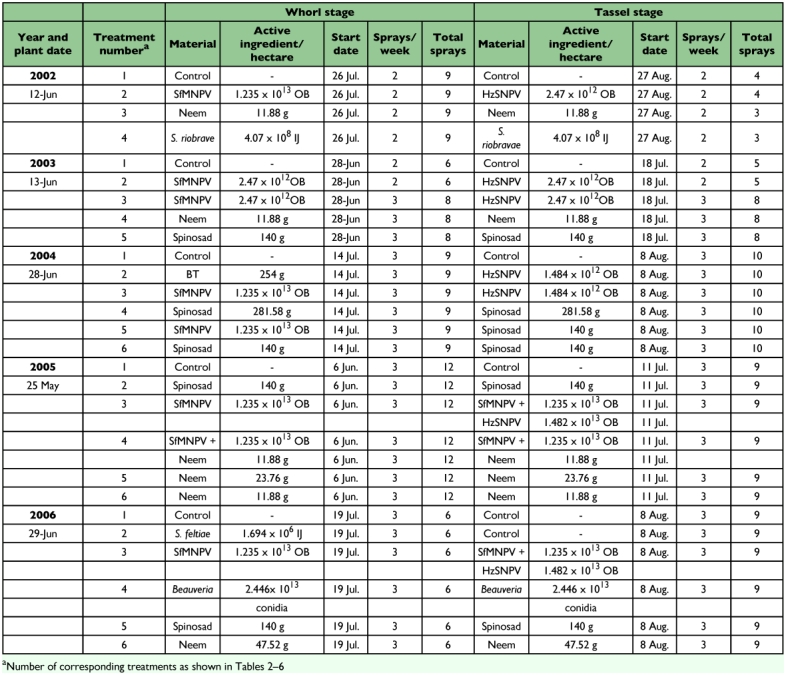
Spray treatments applied to BT transgenic and nontransgenic sweet corn to control lepidopterous pests, Charleston, SC, 2002 – 2006.

There was a significant interaction between corn genotype and spray treatment in 14 of 34 analyses. In analyses of variables most directly related to the fall armyworm (number per plant or ear, whorl stage damage, or plant height), there was a significant interaction in 7 of 19 analyses. Of the variable most related to the corn earworm (number per ear), the interaction was significant in two of five analyses. There was a significant interaction in 5 of 10 of analyses of numbers of ears per sample and ear damage.

Virus treatments reduced densities of fall armyworms on whorl stage corn in 80% of the comparisons with the controls; however, the virus only reduced whorl stage damage in 25% of these comparisons. In no case were there more larvae or damage on virus treatments. SfMNPV improved control of fall armyworms on both Bt and parent corn. This virus application prevented fall armyworms from reducing plant height at the whorl stage on Bt and parent corn in 2002 and on parent corn in 2006. It resulted in reduced whorl stage damage on Bt corn in 2004, and on the parent corn in 2003 and 2005. SfMNPV reduced the number of fall armyworms per whorl stage plant on both Bt and parent corn in 2002, 2003, 2004, and 2006, and reduced the number of larvae per ear on both corn genotypes in 2006. At harvest, the virus treatment prevented reduction in the number of ears per sample on both genotypes in 2002 and on only the parent corn in 2006. Percentage of ears damaged was reduced by the virus treatments on Bt corn in 2005, but was otherwise not affected by virus treatments. HzSNPV reduced the number of corn earworms per ear on Bt and parent corn in 2003, and on only parent corn in 2006.

**Table 2.**
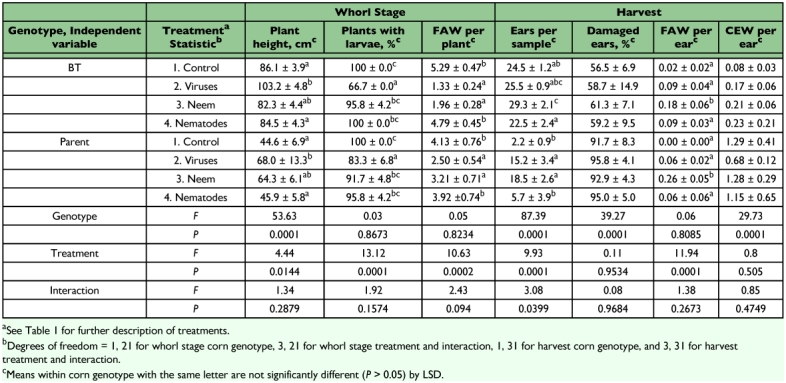
Damage to, and populations of lepidopterous larvae on, BT transgenic and nontransgenic (parent) sweet corn treated with biologically based insecticides, Charleston, SC, 2002.

Neem reduced populations of and/or damage by fall armyworms in several tests. It reduced whorl stage damage on parent corn in 2005 and reduced the number of fall armyworms per plant on both corn genotypes in 2002 and 2006. Neem prevented reduction in the number of ears per sample on Bt and parent corn in 2002, and on the parent corn in 2006, and reduced percent damage to ears only on Bt corn in 2005 and 2006. Relative to the control, there were fewer fall armyworms per ear on neem treatments on both Bt and parent corn in 2005 and 2006, but more fall armyworms per ear on neem treatments on both corn genotypes in 2002 and on the parent corn in 2003. Neem had no effect on the number of corn earworms per ear in any year.

Spinosad consistently reduced populations of larvae and damage of both insect species. It reduced whorl stage fall armyworm densities on both Bt and parent corn in 2003, 2004, and 2006. It also reduced whorl stage damage on Bt corn in 2004 and 2006, and to parent corn in all years in which it was tested. Spinosad reduced ear damage on both Bt and parent corn in all years in which it was tested. Numbers of fall armyworm larvae per ear were reduced by spinosad on both genotypes of corn in all years except on the parent corn in 2003. Numbers of corn earworms per ear were reduced on both Bt and parent corn in 2003 and 2005.

*Beauveria*, tested only in 2006, reduced the number of fall armyworm larvae per plant at whorl stage on both genotypes of corn, but had no other effect. Spray treatments of Bt at whorl stage in 2004 reduced fall armyworms per plant on both corn genotypes but otherwise had no effect. The nematode treatment with *S. feltiae* in 2006 had fewer fall armyworms per whorl stage plant but more fall armyworms per ear at harvest, although the nematodes were not applied to tassel stage corn. Nematode treatment with *S. nobrave* had no observable effect in 2002.

**Table 3.**
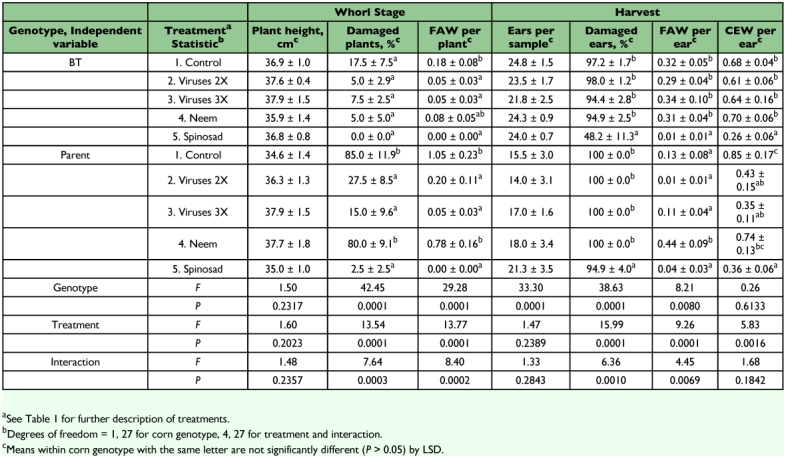
Damage to, and populations of lepidopterous larvae on, BT transgenic and nontransgenic (parent) sweet corn treated with biologically based insecticides, Charleston, SC, 2003.

## Discussion

In the present study, Bt transgenic sweet corn suffered less damage and supported lower densities of fall armyworms and corn earworms than did conventional sweet corn. However, under high pest pressure, as was the case in most of the tests reported herein (due largely to late planting), considerable damage can occur. In these situations, biologically-based spray treatments have the potential to improve insect control.

SfMNPV reduced whorl stage densities of the fall armyworm relative to the control in 80% of comparisons, although whorl stage damage was reduced in only 25% of comparisons. The lesser effect on damage is probably due to high pest pressure; though reduced in numbers, there were still enough larvae to cause plants to be classified as damaged. Previous studies of the fall armyworm showed that the effect of SfMNPV was greater on nontransgenic corn than on Bt corn (Farrar et al. 2004, 2005). In the present study, reductions in densities of fall armyworms were seen on both genotypes of corn, and there were no significant interactions of spray treatment by corn genotype in most analyses of these variables. There were thus no obvious reductions in efficacy of this virus on Bt corn that might preclude its application to this crop. Neem also reduced fall armyworm densities and damage in a number of cases, but increased numbers of larvae were seen in some cases. The most consistent control of the fall armyworm, though, was obtained with spinosad. Spinosad was not compared to a pyrethroid insecticide. However, Lance and All (2005) found that spinosad gave control of fall armyworms on sweet corn comparable to that given by a pyrethroid.

Previous work showed that the corn earworm is more susceptible than the fall armyworm to Cry1Ab toxins expressed by transgenic field corn or by transgenic sweet corn (Farrar et al. 2004, and references cited therein). Nevertheless, significant populations of corn earworms were found on the transgenic corn in our tests in some years, particularly 2003. HzSNPV had limited effects; it reduced the number of corn earworms per ear on both genotypes of corn in 2003 and on parent corn in 2006, but otherwise did not affect this variable. Neem had no effect on corn earworms per ear in any year. As with the fall armyworm, the greatest reduction in corn earworm densities was caused by spinosad, with reductions on both genotypes. Ear damage was consistently reduced by spinosad, although numbers of corn earworms per ear were reduced in only two of the four years in which spinosad was tested. Previous reports have shown spinosad to provide control of corn earworms on sweet corn comparable to that given by pyrethroid insecticides (Cordero et al. 2004; Lance and All 2004, 2005; Edelson and Mackey 2006).

**Table 4.**
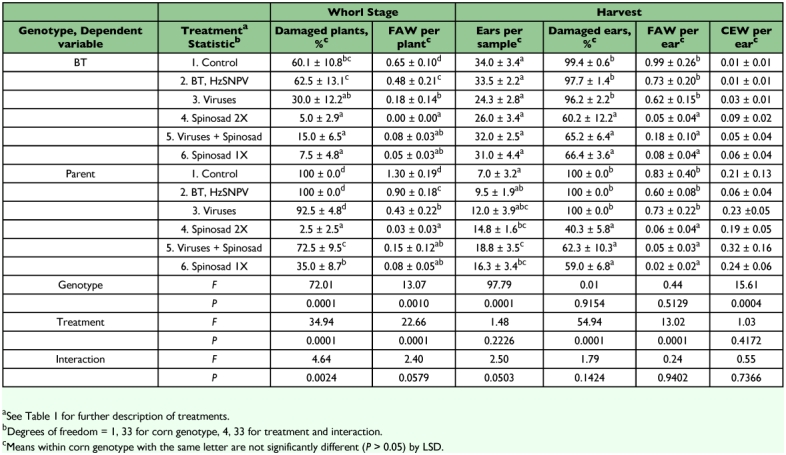
Damage to, and populations of lepidopterous larvae on, BT transgenic and nontransgenic (parent) sweet corn treated with biologically based insecticides, Charleston, SC, 2004.

Some of the treatments that were included in only one year, including the two species of nematodes, *B. bassiana*, and sprayable Bt, showed some effects, but these effects were very limited, and did not include reduced damage. Therefore, we considered these treatments unlikely to provide adequate levels of control in the system that we studied, and so they were not tested further.

The corn for our tests was planted late (May to June) each year to ensure infestations of both fall armyworms and corn earworms. Given the high pest pressure that resulted, it is not surprising that high levels of damage were seen on most treatments. However, we believe that relative differences among the treatments in our study are indicative of which treatments should perform best under lower pest pressure. The recommended time for planting sweet corn in coastal South Carolina is February to March. If the corn were planted earlier in the season, infestations could be expected to be lighter, though they could be problematic in any given year. In cases of light infestations, Bt transgenic corn might not need supplemental control for corn earworm. If fall armyworm, to which Bt sweet corn is less resistant, were present, supplemental control with a biologically-based spray treatment might be adequate to prevent significant losses. Of those treatments evaluated in the present study, spinosad would be the most likely to provide acceptable levels of additional control of both pest species. Among the other materials, SfMNPV has potential utility against the fall armyworm on whorl stage corn.

Further testing of these materials under conditions more representative of growers' fields (e.g., early planting) would be necessary to determine whether the treatments that showed statistically significant effects in the present study can provide control that would be adequate from a grower's standpoint. Such testing would also be necessary to determine cost effectiveness relative to chemical insecticides. Additional factors that may influence acceptability of biologically based controls to growers include limited availability of insecticides for vegetable crops such as sweet corn and the potential development of resistance to these insecticides. These factors could make biologically based insecticides more attractive as supplemental controls for lepidopterous pests on transgenic sweet corn.

**Table 5.**
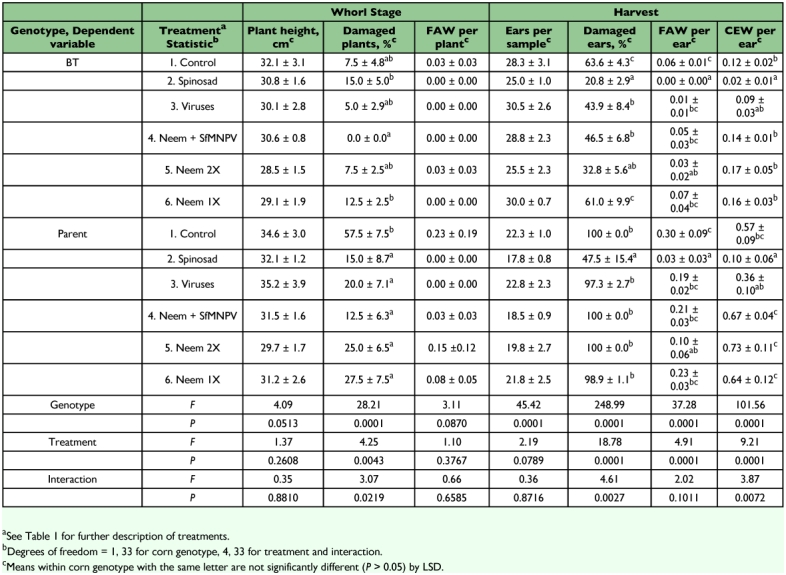
Damage to, and populations of lepidopterous larvae on, BT transgenic and nontransgenic (parent) sweet corn treated with biologically based insecticides, Charleston, SC, 2005.

**Table 6.**
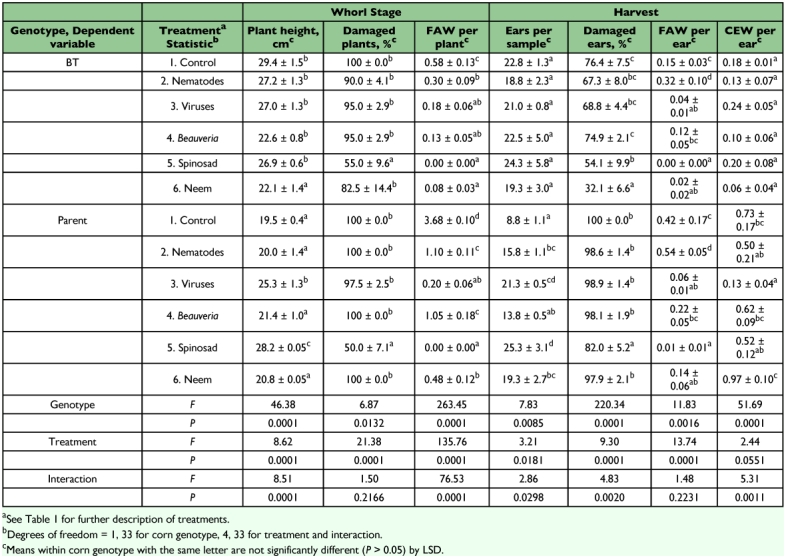
Damage to, and populations of lepidopterous larvae on, BT transgenic and nontransgenic (parent) sweet corn treated with biologically based insecticides, Charleston, SC, 2006.

## Abbreviations

Bt: *Bacillus thuringiensis*
CryIAb: endotoxin from Bt
HzSNPV: *Helicoverpa zea* singly-embedded nucleopolyhedrovirus
NPV: nucleopolyhedrovirus
SfMNPV: *Spodoptera frugiperda* multiply-embedded nucleopolyhedrovirus
